# A review of the dietary diversity and micronutrient adequacy among the women of reproductive age in low‐ and middle‐income countries

**DOI:** 10.1002/fsn3.3855

**Published:** 2023-11-20

**Authors:** Md. Hafizul Islam, Md. Moniruzzaman Nayan, Ahmed Jubayer, Md. Ruhul Amin

**Affiliations:** ^1^ Institute of Nutrition and Food Science, University of Dhaka Dhaka Bangladesh; ^2^ Inspira Advisory and Consulting Limited Dhaka Bangladesh; ^3^ Bangladesh Institute of Social Research (BISR) Trust Dhaka Bangladesh

**Keywords:** dietary diversity, low‐ and middle‐income countries, micronutrient intake, women of reproductive age

## Abstract

The dietary quality of women of reproductive age (WRA) is particularly important during preconception, conception, and pregnancy for themselves and their offspring. Poorly diversified diets resulting in inadequate micronutrient consumption may have adverse effects on their health. This narrative review summarizes the findings of studies reporting on dietary diversity and micronutrient intake by WRA in low‐ and middle‐income countries (LMICs). Studies on WRA aged 15–49 years in LMICs, with a sample size of more than 150, report dietary diversity and multiple micronutrient intake based on 24‐h dietary recall/food weighed record/food frequency questionnaire, and published between January 2011 and June 2021 were included. The results were compared to the Food and Agriculture Organization (FAO) recommended cut‐off for dietary diversity and the Indian Council of Medical Research (ICMR) recommended age‐ and sex‐specific estimated average requirements (EARs) for micronutrient intake. This review includes 35 articles, of which 21 focused on dietary diversity and 14 on micronutrient intake. The results showed that WRA in LMICs had inadequate dietary diversity, with mean food group consumption of only 3.0–4.84, and around 42.3%–90% of women consumed inadequately diversified diets (<5 food groups). Additionally, most studies found that WRA did not consume adequate amounts of essential micronutrients, particularly calcium, iron, zinc, vitamin A, thiamin, riboflavin, folate, and vitamin B_12_. However, the intake of vitamin C, niacin, and vitamin B_6_ was above the required levels. In conclusion, this review highlights the common inadequacy of dietary diversity and multiple micronutrient intake among WRA in most LMICs. Effective measures involving improving dietary diversity, food fortification with micronutrients, and supplementation programs could help improve the dietary quality and intake of optimal micronutrients by women in LMICs.

## Graphical abstract

WRA in LMICs had inadequate dietary diversity, with mean food group consumption of only 3.0–4.84, and around 42.3%–90% of women consumed inadequately diversified diets (<5 food groups). Most studies found that WRA did not consume adequate amounts of essential micronutrients, particularly calcium, iron, zinc, vitamin A, thiamin, riboflavin, folate, and vitamin B12.

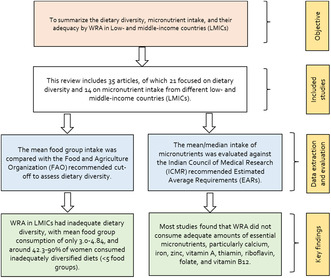

## 1 INTRODUCTION

The nutritional and health condition of reproductive‐aged women is determined by food quality, as well as different sociodemographic factors including food security, literacy, sanitation, and healthcare facility (Allen et al., 2009; Milton et al., 2010; Mtumwa et al., 2016; UNICEF, 2021; Waghmare et al., 2022). Dietary quality is defined from both qualitative and quantitative aspects (Gil et al., 2015; Wirt & Collins, 2009). The qualitative approach reflects the dietary patterns and diversity in food group choices. The Food and Agriculture Organization (FAO) recommends consuming at least five different food groups for women of reproductive age (WRA) (FAO, 2016). Inadequate diversified diet among WRA can have serious consequences for their overall health and well‐being, as well as their ability to conceive and carry a healthy pregnancy. Poor dietary intake and inadequate diversified diets during pregnancy contribute to maternal malnutrition in LMICs (Lander et al., 2019; Lee et al., 2013). Evidence substantially demonstrates that when a mother is malnourished, her children are at risk for low birth weight, stunting, long‐term cognitive impairment, delayed mental development, and neonatal death (Black et al., 2008; Christian et al., 2015; Lassi et al., 2020). It is suggested that high‐quality, diverse diets are an effective way of providing an adequate amount of micronutrients (Martin‐Prevel et al., 2015). Women in resource‐poor regions frequently consume low‐quality meals consisting mostly of cereals, which exposes them to a significant risk of lacking adequate micronutrients (Torheim et al., 2010). Moreover, consumption of a diverse diet leads to a healthy life, which may also prevent many non‐communicable diseases like type 2 diabetes, coronary heart disease, etc. (Montonen et al., 2005; Osganian et al., 2003). Micronutrient intakes, such as vitamins C and E, may lessen the risk of cardiovascular disease among women (Osganian et al., 2003). As a result, for long‐term health and well‐being, a balanced diet containing an adequate amount of nutritional components is required.

The quantitative aspect of diet quality includes the dietary nutrient intake and its adequacy aligned with the recommended value. A diet that is low in essential vitamins and minerals, such as iron, folic acid, and calcium, can increase the risk of iron deficiency anemia and osteoporosis, which can lead to maternal morbidity and mortality (Qadir et al., 2022). Around the world, WRA (both pregnant and non‐pregnant) from LMICs, are at high risk of micronutrient deficiency, also referred to as hidden hunger. Insufficient micronutrient intake and predominantly plant‐based diets during pregnancy have been mentioned among women from LMICs in Asia, Africa, and Latin America (Lee et al., 2013). The intake of several micronutrients, particularly folate, iron, calcium, and zinc, was inadequate among the women (Lee et al., 2013). A review of industrialized countries (the United States/Canada, the United Kingdom, Europe, Australia/New Zealand, and Japan) revealed the same picture, with pregnant women's diets failing to satisfy national dietary intake requirements for folate, iron, vitamin D, or calcium (Blumfield et al., 2013).

It is recommended that WRA must maintain good health status during preconception, conception, and pregnancy for themselves and for the betterment of their children through safe pregnancy, safe delivery, and better pregnancy outcome. They have unique nutrient needs to support overall health and well‐being, as well as to prepare for potential pregnancy. Adequate intake of nutrients such as iron, calcium, and vitamin D is important for maintaining healthy bones, as well as preventing iron deficiency anemia (Qadir et al., 2022). Adequate intake of folic acid is also important for WRA, as it can help prevent certain birth defects if taken before pregnancy (Bibbins‐Domingo et al., 2017). Additionally, adequate intake of vitamin B_12_ is important for maintaining healthy blood cells and proper nerve function (Al‐Musharaf et al., 2021). In addition, to meet the extra demands of pregnancy and lactation, women must enter their pregnancy with optimal micronutrient status. It is unfortunate when a previous review indicated that above 50% of pregnant women in developing nations and 42% globally are anemic, primarily because of dietary iron deficiency (Bailey et al., 2015). In addition, many people consume insufficient amounts of other important vitamins and minerals, such as vitamin A, folate, calcium, and zinc.

Previous reviews available in the literature primarily focused on the dietary micronutrients intake of pregnant women from both developing countries (Harika et al., 2017; Lee et al., 2013; Torheim et al., 2010) and developed countries (Bellows et al., 2020). However, no study has yet summarized dietary diversity and micronutrient intake among WRA from LMICs during the last decade. In this review, we have summarized the published findings on the dietary intake of WRA of LMICs, excluding pregnant and lactating women. It is anticipated that this review will provide a better insight into the dietary diversity and micronutrient adequacy of WRA of LMICs.

## 2 METHODS

### 2.1 Literature search strategy

Relevant studies on the dietary diversity and dietary micronutrient consumption of WRA from LMICs published between January 2011 and June 2021 in peer‐reviewed journals were identified through an exhaustive literature search on electronic databases: PubMed and Google Scholar. Relevant keywords and Medical Subjects Headings (MeSH) terms were used for the literature search. The search strategy for PubMed database has been given in Box 1. Moreover, a manual search on Google and a reference list screening of the included studies were done. The literature search was conducted in June–July 2022.

BOX 1 **Search strategy for PubMed database.**

*Non‐pregnant and non‐lactating women*

1. ‘Women of reproductive age’ OR ‘Non‐pregnant and non‐lactating women’ OR ‘Women of childbearing age’ OR ‘Female’ OR ‘Mother’

*Dietary diversity*

2 ‘Dietary diversity’ OR ‘Minimum dietary diversity’ OR ‘Women dietary diversity’ OR ‘Dietary quality’ OR ‘Dietary patterns’

*Micronutrients intake*

3 ‘Dietary intake’ OR ‘Dietary nutrient intake’ OR ‘Micronutrient intake’ OR ‘Dietary adequacy’ OR ‘Nutrients adequacy’ OR ‘Vitamins’ OR ‘Minerals’

*Low‐ and middle‐income countries*

4 ‘Low‐ and middle‐income countries’ OR ‘Low‐income countries’ OR ‘Poor countries’ OR ‘Developing countries’ OR ‘Underdeveloped countries’ OR ‘Asia’ OR ‘Africa’ OR ‘Latin America’
5 #1 AND #2 AND #4
6 #1 AND #3 AND #4

### 2.2 Eligibility criteria

The studies which reported the dietary diversity and micronutrient intake of WRA (15–49 years) from LMICs were included in this review. Two authors independently screened the titles, abstract, and full‐text documents. Any disagreement between the two authors was discussed to reach a consensus. Regarding troubled documents, an agreement was reached by the other researcher. LMICs from Africa, Asia, and Latin America defined by the World Bank were included here (World Bank, 2020). These studies were from peer‐reviewed journals published in English from January 2011 to June 2021. The method of the dietary assessment was also considered; only studies that used a 24‐h recall weighted or estimated records or food frequency questionnaire (FFQ) were considered suitable for inclusion. The studies with a sample size of <150 and those reported micronutrient supplementation were excluded. Moreover, the studies that reported WRA with NCDs or any other complications were excluded. Studies reporting women's dietary diversity based on minimum dietary diversity for WRA (MDD‐W) and/or Women's dietary diversity score (WDDS) guidelines were included in the review process for dietary diversity. The full‐text studies obtained from the literature search were evaluated against the eligibility criteria summarized in Table 1.

**TABLE 1:** Eligibility criteria for inclusion of studies in the review.

Study characteristics	Methods	Dietary diversity	Micronutrients
Cross‐sectional studies Non‐pregnant and non‐lactating women (15–49 years) A sample size of >150 Published between January 2011 and June 2021 Conducted in LMICs	Single/multiple days 24‐h recall weighed/estimated record/FFQ Reporting micronutrients intake in mean/median Minimum dietary diversity for women (MDDW) or dietary diversity score of women (WDDS)	MDDW/WDDS score Prevalence of adequacy of dietary diversity	Vitamin A Vitamin C Thiamin Riboflavin Niacin Vitamin B_6_ Folate Vitamin B_12_ Iron Zinc Calcium

### 2.3 Assessment of dietary diversity

Information on the method of the studies, key findings, including the number of food groups consumed, and the rate of the adequacy of minimum dietary diversity was extracted from the studies based on a predetermined data extraction form using Microsoft Excel 2016. Consumption of FGs was compared against the recommended cut‐off value of five FGs (FAO, 2016). This review has also reported information on the percentage of women consuming adequate diversified diets.

### 2.4 Assessment of dietary micronutrient intake

Per‐day dietary micronutrient intake by WRA in LMICs has been summarized in this review. The mean (standard deviation, SD) or median (interquartile range, IQR) of various micronutrients (iron, calcium, zinc, vitamin A, vitamin C, thiamin, riboflavin, niacin, pyridoxine, folate, and vitamin B_12_) has been extracted from the studies. These values were summarized as range (min–max intake) and later compared with the recommended intake value for assessing the adequacy of micronutrient intake. Age‐ and sex‐specific estimated average requirements (EARs) were used as a cut‐off for determining the adequacy of intake by WRA. These recommended cut‐off values were adopted from the Indian Council of Medical Research (ICMR) (ICMR‐NIN, 2020).

## 3 RESULTS

### 3.1 Dietary diversity among women of reproductive age in LMICs

**FIGURE 1:**
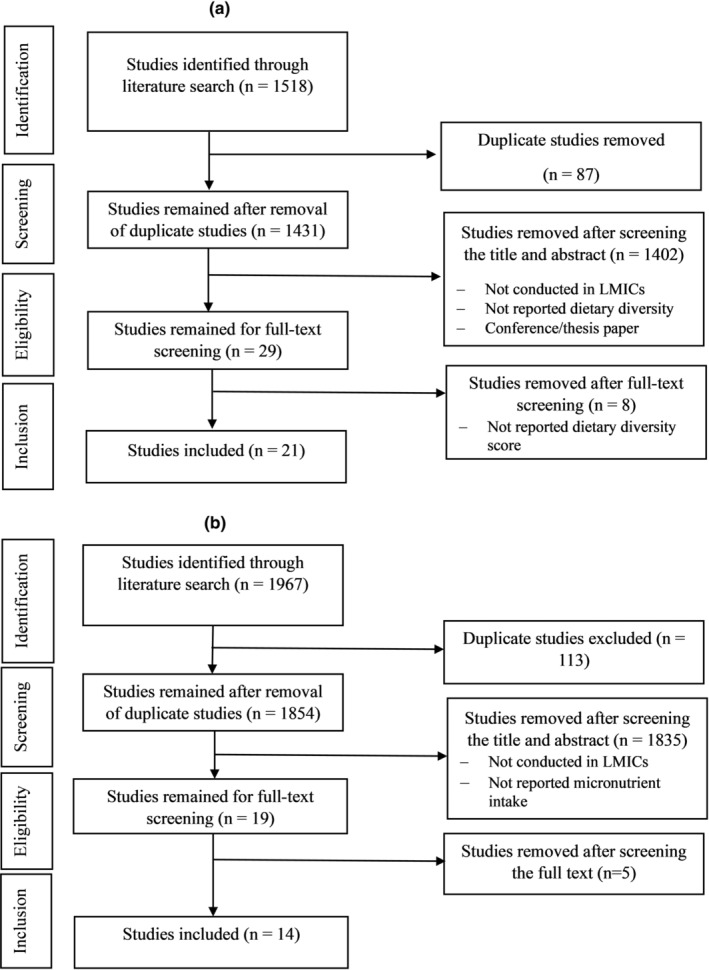
Flowchart of the study selection process of (a) dietary diversity; (b) micronutrients intake.

The study selection process for dietary diversity has been demonstrated in Figure 1a. A total of 1518 studies were identified through PubMed and Google Scholar database searches. First, 87 duplicate articles were excluded. After screening the title and abstract, 1402 studies were excluded. These excluded studies were not conducted in LMICs and did not report the dietary diversity of women. After screening the full text, eight studies not meeting the selection criteria were excluded (Figure 1a). Finally, 21 studies reporting dietary diversity were selected for this review. Dietary diversity among the WRA from LMICs has been summarized in Table 2. These studies were conducted in different LMICs (Mali, Kenya, Ghana, Bangladesh, Sri Lanka, Uganda, Tanzania, Burkina Faso, Zambia, India, Niger, Myanmar, Latin America, and Ethiopia). The studies included 47,579 WRA (range 180–12,754) with a mean age of 29.8 years. All the studies collected 24‐h dietary recall data while a study in Tanzania (Bellows et al., 2020) used a food frequency questionnaire (FFQ) for the last 1 month to collect dietary data. Almost all the studies measured dietary diversity using FAO‐recommended 10 FGs except the study in Niger (Cisse‐Egbuonye et al., 2017). The identified studies with FG consumption data showed that the mean intake of FGs was 3–4.84 of 10, indicating inadequate diversity compared to the recommendation (at least five different FGs).

**TABLE 2:** Dietary diversity among the women of reproductive age in low‐ and middle‐income countries.

Author, year	Country	Study population	Sample size	Dietary data collection methods	Methods of dietary diversity assessment	Age	Number of food groups consumed	Prevalence of adequate dietary diversity (%)
Adubra et al. (2019)	Mali, West Africa	WRA	4995	Single 24‐h recall	MDD‐W	28.56 (0.13)	3.82 (0.05)	27
Gitagia et al. (2019)	Kenya	WRA	384	Single 24‐h recall	MDD‐W	29.0	3.81	22
Amugsi et al. (2016)	Ghana	WRA	2262	Single 24‐h recall	MDD‐W	30.10 (7.02)	–	43.1
Sinharoy et al. (2018)	Bangladesh	WRA	2599	Single 24‐h recall	MDD‐W	24.7	3.87	31
Ekesa et al. (2020)	Uganda	WRA	1122	Single 24‐h recall	MDD‐W	29.6 (7.81)	–	53
Weerasekara et al. (2020)	Sri Lanka	WRA	400	Single 24‐h recall	MDD‐W	–	–	36.75
Chakona & Shackleton (2017)	South Africa	WRA	554	48‐h recall	MDD‐W	32 (10)	3.46 (0.99)	25
Bellows et al. (2020)	Tanzania	WRA	1006	FFQ of 1 months	MDD‐W	–	3.0 (1)	10
Custodio et al. (2020)	Burkina Faso	WRA	12,754	Single 24‐h recall	MDD‐W	–	3.8	31
Kaliwile et al. (2019)	Zambia	WRA	530	Single 24‐recall	MDD‐W	27.6 (8.7)	3.75 (1.11)	23.3
Gupta et al. (2020)	India	Households	3600	Single 24‐h recall	MDD‐W	–	4.28 (1.85)	–
Cisse‐Egbuonye et al. (2017)	Niger	WRA	3360	Single 24‐h recall	WDDS 9 FG	29.63 (8)	3.5	–
Rammohan et al. (2019)	Myanmar	Households	3230	Single 24‐h recall	MDD‐W	–	4.09	–
Diop et al. (2021)	Burkina Faso	WRA	432	Single 24‐h recall	MDD‐W	31 (6.6)	3.3 (1.2)	–
Harris‐Fry et al. (2015)	Bangladesh	WRA	2620	Single 24‐h recall	WDD‐S	30.8 (8.0)	3.8	–
Arsenault et al. (2013)	Bangladesh	WRA	478	24‐h recall on 2 nonconsecutive days	DDS 9 FG	27.9 (8.0)	4.3 (0.9)	–
Sultana et al. (2019)	Bangladesh	WRA	180	24‐h recall on 3 nonconsecutive days	DDS 9 FG	–	4.84 (1.02)	–
Gómez et al. (2020)	Latin America	WRA	3704	24‐h recall on 2 nonconsecutive days	MDDW	–	4.73 (1.34)	57.7
Pal et al. (2017)	India	WRA	182	Single 24‐h recall	MDDW	33.1	–	46.2
Abraham et al. (2015)	Ethiopia	WRA	284	Single 24‐h recall	WDDS	28.5 (8.97)	4.3 (1.05)	42.6
Wable Grandner et al. (2021)	Bangladesh	WRA	2903	Single 24‐h recall	WDDS	34.7 (7.5)	3.73 (0.44)	–
Total/Range			47,579			29.8 (2.49)	3.0–4.84	10–50.7

Abbreviations: FFQ, Food Frequency Questionnaire; MDD‐W, Minimum Dietary Diversity for Women; WDDS, Women Dietary Diversity Score; ages and food groups consumption were presented as mean (SD)/median (IQR); WRA, Women of Reproductive Ages (15–49 years).

None of the studies could meet the adequacy of diversity in diets. A study among female residential university students in Bangladesh showed the highest number of FGs (4.84) consumption (Sultana et al., 2019), while the women of Tanzania (Bellows et al., 2020) consumed less number of FGs (3.0). The prevalence showed that, on average, only 10–50.7% of the women consumed adequately diversified diets. The rate of adequacy was lowest in Tanzania (10%) (Bellows et al., 2020) and highest in Uganda (53%) (Ekesa et al., 2020).

### 3.2 Dietary micronutrient intake by the WRA in LMICs

The study selection process for the micronutrient intake has been presented in Figure 1b. A total of 1967 studies were identified through PubMed and Google Scholar database searches. First, 113 duplicate articles were excluded. After screening the title and abstract, 1835 studies were excluded. After screening the full text, five studies not met the selection criteria were excluded. Finally, 14 studies were selected for this review.

**FIGURE 2:**
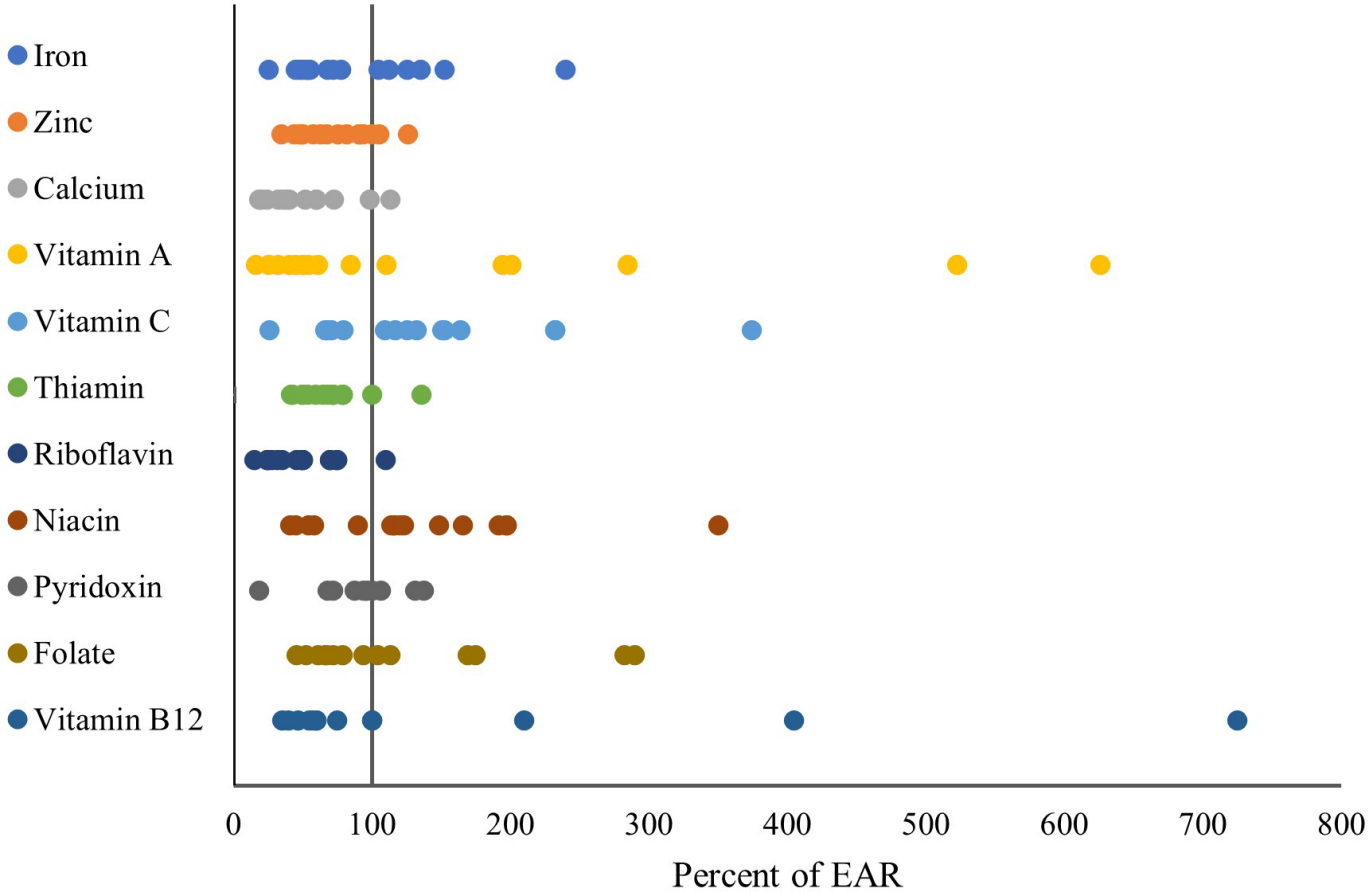
Intake of micronutrients among WRA presented as a percentage of EAR. The circle in this figure represents the mean intake of nutrients in each study as compared to the EAR of respective nutrient.

Dietary micronutrient intake by WRA in LMICs has been summarized in Table 3. The studies were conducted in different LMICs (India, Vietnam, Congo, Ethiopia, South Africa, Bangladesh, Nigeria, Brazil, Lebanon, and Zambia). All the studies collected 24‐h recall dietary data while two studies in India (Nunn et al., 2019) and Vietnam (Nguyen et al., 2014) used FFQ to collect dietary data. These studies included 29,573 WRA (range 152–11,029) with a mean age of 28.51 years. The intake of various micronutrients among WRA has been illustrated as the percentage of EAR in Figure 2.

**TABLE 3:** Dietary micronutrient intake by the women of reproductive age in low‐ and middle‐income countries.

Author, year country	Dietary data collection method	Sample size	Age	Iron (mg)	Zinc (mg)	Calcium (mg)	Vitamin A (μg)	Vitamin C (mg)	Thiamin (mg)	Riboflavin (mg)	Niacin (mg)	B_6_ (mg)	Folate (μg)	B_12_ (μg)
Nunn et al. (2019) India	7 days FFQ	6238	–	7.9 (3.9)	5.24 (2.19)	293 (177)	196 (159)	37.3 (28.1)	0.75 (0.36)	0.5 (0.30)	6.99 (4.55)	1.15 (0.48)	130 (58)	1.15 (1.0)
Nguyen et al. (2014) Vietnam	3 months FFQ	4983	26.2 (4.6)	15.7 (8.3)	10.0 (4.1)	–	783 (721)	206 (182)	1.4 (0.8)	1.0 (0.8)	17.8 (9.8)	1.5 (0.7)	304 (218)	1.5 (1.8)
Moumin et al. (2020) Congo	Single 24‐h recall	444	29.2 (0.02)	18.8 (0.3)	8.3 (0.2)	581 (11)	757 (14)	83 (1)	0.95 (0.02)	0.98 (0.02)	14.4 (0.2)	2.1 (0.03)	522 (13)	14.5 (12)
Moumin et al. (2020) Congo	Single 24‐h recall	300	29.9 (0.02)	20.3 (0.5)	9.0 (0.3)	789 (20)	1109 (19)	90 (1)	1.0 (0.02)	1.0 (0.03)	13.7 (0.3)	1.6 (0.04)	508 (15)	2.0 (0.2)
Asayehu et al. (2017) Ethiopia	Single 24‐h recall	164	30.4 (5.4)	36.0	7.4	302.6	2037.9	83.7	0.6	2.2	6.5	–	144.5	–
Oldewage‐Theron and Kruger (2011) South Africa	Single 24‐h recall	426	–	3.8 (2.0)	3.8 (2.4)	150.5 (176.8)	176.3 (617.9)	14.4 (14.9)	14.4 (14.9)	0.3 (0.3)	4.9 (4.1)	0.3 (0.2)	81.9 (103.6)	1.2 (3.2)
Islam et al. (2021) Bangladesh	Single 24‐h recall	355	–	11.63	13.84	258.42	98.66	64.30	1.90	0.91	42.0	–	187.25	–
Arsenault et al. (2013) Bangladesh	24‐h recall on 2 nonconsecutive days	235	27.9 (8.0)	8.3 (2.0)	5.4 (1.1)	194 (86)	239 (167)	60 (27)	0.9 (0.2)	0.5 (0.1)	23.0 (4.0)	2.2 (0.4)	94.0 (34.0)	0.7 (0.5)
Akter et al. (2021) Bangladesh	Single 24‐h recall	3886	–	10.8	10.3	323.6	126.7	72.8	1.1	0.7	19.9	1.7	168.7	0.8
Sultana et al. (2019) Bangladesh	Single 24‐h recall	180	–	6.72	6.35	161.56	157.23	38.79	0.83	0.63	10.77	–	109.93	0.93
De Moura et al. (2015) Nigeria	Single 24‐h recall	579	28.2 (7.9)	16.8	11	481	2441	–	–	–	–	1.54	315	8.1
Barbosa (2013) Brazil	Single 24‐h recall	11,029	–	10.2	10	477	431	127.9	1.1	1.5	23.7	1.4	–	4.2
Abou‐Rizk et al. (2021) Lebanon	Single 24‐h recall	152	28.7 (6.1)	8.0 (6.1)	5.5 (3.5)	414.1 (325.9)	329.8 (397.3)	52.9 (43.0)	1.1 (0.6)	0.9 (0.5)	11.9 (6.9)	0.8 (0.5)	203.8 (178.3)	1.2 (1.8)
Kaliwile et al. (2019) Zambia	Single 24‐h recall	343	27.6 (8.7)	7.39	6.93	146	210	43.8	0.58	0.55	14.8	1.0	121	0.00
Loukrakpam et al. (2020) India	Single 24‐h recall	259	–	22.83	11.55	905	63.61	68.99	1.01	1.39	13.96	1.53	418	–
Total/Range		29,573	28.51 (1.34)	3.8–36.0	3.8–13.84	146–905	63.61–2037.9	14.4–206	0.58–14.4	0.3–2.2	4.9–42.0	0.3–2.2	81.9–522	0.7–14.5

*Note*: Age and amount of micronutrients intake were presented as mean (SD)/median (IQR). Vitamins and minerals intake were summarized as range.

Abbreviation: FFQ, Food frequency questionnaire.

#### 3.2.1 Iron

The intake of daily dietary iron by the women in LMICs (3.8–36.0 mg) was above the daily EAR of 15 mg. The studies in Ethiopia (36.0 mg) (Asayehu et al., 2017), Vietnam (15.5 mg) (Nguyen et al., 2014), Congo (18.8–20.3 mg) (Moumin et al., 2020), Nigeria (16.8 mg) (De Moura et al., 2015), and India (22.3 mg) (Loukrakpam et al., 2020) indicated that dietary iron intake was satisfactory compared to the EAR. On the other hand, studies in Lebanon (8.0 mg) (Abou‐Rizk et al., 2021), India (7.39 mg) (Nunn et al., 2019), Zambia (7.39 mg) (Kaliwile et al., 2019), and South Africa (3.8 mg) (Oldewage‐Theron & Kruger, 2011), and Bangladesh (6.72–11.63 mg) (Akter et al., 2021; Arsenault et al., 2013; M. H. Islam et al., 2021; Sultana et al., 2019) showed lower intake of iron than the recommended EAR.

#### 3.2.2 Zinc

Daily mean zinc intake by women was 3.8–13.84 mg, while the EAR is 11 mg. Most of the studies showed lower intake of dietary zinc as compared to EAR, especially in India (5.24 mg) (Nunn et al., 2019), South Africa (3.8 mg) (Oldewage‐Theron & Kruger, 2011), Lebanon (5.5 mg) (Abou‐Rizk et al., 2021), Ethiopia (7.4 mg) (Asayehu et al., 2017), Zambia (6.93 mg) (Kaliwile et al., 2019), and Bangladesh (5.4 mg) (Arsenault et al., 2013) as compared to the EAR.

#### 3.2.3 Calcium

All the studies included in this review indicated that dietary calcium intake (146–905 mg) was very poor among the women in LMICs compared to their EAR (800 mg). The intake of calcium was excessively lower among women of Zambia (146 mg) (Kaliwile et al., 2019), and South Africa (150.5 mg) (Oldewage‐Theron & Kruger, 2011), Ethiopia (302.6 mg) (Asayehu et al., 2017), and Bangladesh (161.56–323.6 mg) (Akter et al., 2021; Arsenault et al., 2013; Islam et al., 2021; Sultana et al., 2019). Only a study in India (905 mg) (Loukrakpam et al., 2020) showed adequate intake of calcium among the women.

#### 3.2.4 Vitamin A

Per day intake of vitamin A by WRA in LMICs was 63.61–2441 μg. Although the consumption of vitamin A was inadequate in most of the studies as compared to the EAR (390 μg), the studies in Vietnam (783 μg) (Nguyen et al., 2014), Congo (757–1109 μg) (Moumin et al., 2020), Ethiopia (2037.9 μg) (Asayehu et al., 2017), and Nigeria (2441 μg) (De Moura et al., 2015) showed adequate intake of vitamin A among the WRA.

#### 3.2.5 Vitamin C

Daily mean intake of vitamin C by the WRA was 14.4–206 mg. Consumption of vitamin C was adequate in most of the studies compared to the EAR (55 mg). On the contrary, studies in India (37.3 mg) (Nunn et al., 2019), South Africa (14.4 mg) (Oldewage‐Theron & Kruger, 2011), Bangladesh (38.79 mg) (among female residential university students) (Sultana et al., 2019), Lebanon (52.9 mg) (Abou‐Rizk et al., 2021), and Zambia (43.8 mg) (Kaliwile et al., 2019) showed poor dietary intake of vitamin C as compared to the EAR.

#### 3.2.6 Thiamin

Thiamin intake was good in most studies compared to the EAR (1.4 mg). The daily mean intake of thiamin by the WRA was 0.58–14.4 mg which was inadequate compared to the EAR in most of the studies. The intake was excessively low in India (0.75 mg) (Nunn et al., 2019), Ethiopia (0.6 mg) (Asayehu et al., 2017), and Zambia (0.58 mg) (Kaliwile et al., 2019). However, the intake of thiamin among the women of Vietnam (1.4 mg) (Nguyen et al., 2014), and South Africa (14.4 mg) (Oldewage‐Theron & Kruger, 2011) was adequate compared to the EAR.

#### 3.2.7 Riboflavin

The intake of riboflavin was highest in Ethiopia (2.2 mg) (Asayehu et al., 2017) and lowest in a study in South Africa (0.30 mg) (Oldewage‐Theron & Kruger, 2011). Average daily dietary riboflavin intake (0.3–2.2 mg) was inadequate compared to the EAR of 2 mg in almost all the studies. Only the study in Ethiopia (Asayehu et al., 2017) showed adequate intake of riboflavin among the women.

#### 3.2.8 Niacin

Daily mean niacin intake by the women in LMICs was 4.9–42.0 mg which met the daily EAR of 12 mg in most of the countries. However, the women from India (6.99 mg) (Nunn et al., 2019), Ethiopia (6.5 mg) (Asayehu et al., 2017), and South Africa (4.9 mg) (Oldewage‐Theron & Kruger, 2011) showed poor dietary intake of niacin as compared to the EAR.

#### 3.2.9 Pyridoxine

Dietary vitamin B_6_ intake (0.3–2.2 mg) was sufficient in half of the studies compared to the EAR (1.6 mg). The studies in South Africa (0.3 mg) (Oldewage‐Theron & Kruger, 2011), Lebanon (0.8 mg) (Abou‐Rizk et al., 2021), and Zambia (1.0 mg) (Kaliwile et al., 2019) showed excessively poor intake of vitamin B_6_ among women compared to the EAR.

#### 3.2.10 Folate

Average daily dietary folate intake was 81.9–522 μg by the women of LMICs. Most of the studies indicated a low dietary folate intake as per the EAR (180 μg). The women of Congo (508–522 μg) (Moumin et al., 2020), India (418 μg) (Loukrakpam et al., 2020), Vietnam (304 μg) (Nguyen et al., 2014), and Lebanon (203.8 μg) (Abou‐Rizk et al., 2021) showed adequate dietary folate intake compared to the EAR.

#### 3.2.11 Cobalamin

Daily intake of vitamin B_12_ by the women in LMICs was 0.7–14.5 μg while the EAR is 2 μg. Vitamin B_12_ intake was poor in most countries compared to the EAR. However, the studies in Nigeria (8.1 μg) (De Moura et al., 2015), Brazil (4.2 μg) (Barbosa, 2013), and a study in two different regions of Congo (14 μg and 2 μg) (Moumin et al., 2020) showed adequate vitamin B_12_ intake.

## 4 DISCUSSION

This narrative review provides an overall picture of dietary diversity and micronutrient adequacy among WRA in LMICs during the last decade. The mean food group consumption (3.0–4.84) among WRA in LMICs was inadequate compared to FAO's recommendation of consuming at least five FGs. Micronutrient intake among the WRA of LMICs was also poor. Multiple micronutrients, especially calcium, iron, zinc, vitamin A, thiamin, riboflavin, folate, and vitamin B_12_ intake, were inadequate in most LMICs compared to their EARs.

Inadequate dietary diversity among WRA reflects cereal‐based monotonous diet consumption, especially low in fruits, vegetables, and animal foods (poultry, meat, fish, egg, and dairy products) in LMICs. A recent study in four LMICs, Guatemala, India, Pakistan, and Congo also showed inadequately diversified dietary intake among pregnant women (Lander et al., 2019). However, evidence in Bangladesh showed that although dietary diversity has been improved in the last decade irrespective of age and sex, diets remain imbalanced with almost 70% energy come from carbohydrate (Ahmed et al., 2022). Literature suggests that in LMICs, several issues including low production (Harris‐Fry et al., 2015), poverty and food insecurity (Arimond, Wiesmann, Becquey, Daniels, et al., 2010; Brinkman et al., 2010; Harris‐Fry et al., 2015), and lack of nutritional knowledge contributed to inadequate dietary diversity and dependence to cereal‐based diets. Minimum dietary diversity for WRA can also be used as an indication of micronutrient adequacy and hence as a measure of the household access to micronutrient‐rich diet (Arimond, Wiesmann, Becquey, Carriquiry, et al., 2010; FAO, 2010; Martin‐Prevel et al., 2015). Thus, micronutrient deficiencies among WRA in LMICs, especially in Africa and Asia, reflect their inadequate diversity in daily consumption (Allen, 2005).

Inadequate intake of multiple micronutrients in developing countries is typical among NPNL and PLW (M. H. Islam et al., 2023; Islam et al., 2022; Lander et al., 2019; Torheim et al., 2010). Micronutrient deficiencies are estimated to affect more than half of preschool‐aged children and two‐thirds of non‐pregnant women of reproductive age worldwide (Stevens et al., 2022). In addition, more than half (57%) of non‐pregnant women of reproductive age with micronutrient deficiencies live in east and south Asia (Stevens et al., 2022). The findings of the current study also highlighted that the intake of calcium, iron, zinc, vitamin A, thiamin, riboflavin, folate, and vitamin B_12_ was below the EARs in most studies. Moreover, women from South Africa, India, and female residential university students of Bangladesh were low in vitamin C and most of the B vitamins intake (Nunn et al., 2019; Oldewage‐Theron & Kruger, 2011; Sultana et al., 2019). Concurrent inadequacies in multiple micronutrient intake and low dietary diversity justify that diversified diets ensure adequate micronutrients (Martin‐Prevel et al., 2015). In low‐resource settings, the diets of pregnant women are also predominantly plant‐based, with insufficient consumption of nutrient‐rich foods leading to inadequate intake of multiple micronutrients (Arimond, Wiesmann, Becquey, Carriquiry, et al., 2010). Moreover, micronutrient inadequacies, especially vitamin A, iron, folate, and calcium, indicate why micronutrient fortification and supplementation programs have been launched in most LMICs (Keats et al., 2019).

Our results showed a low dietary iron intake, which is consistent with the findings from other studies in both NPNL women and PLW in LMICs (Lander et al., 2019; Torheim et al., 2010). Based on the WHO criteria, a recent analysis concluded that the prevalence of anemia and iron deficiencies in PW and WRA in Ethiopia, Kenya, Nigeria, and South Africa is a public health problem (Harika et al., 2017). Inadequate dietary iron intake and poor dietary quality are contributing factors to the higher prevalence of anemia and iron deficiencies. Thus, epidemiology of iron deficiency anemia is still a public health concern in LMICs (Balarajan et al., 2011). Moreover, iron deficiency is the most common cause of anemia in underprivileged areas. This iron deficiency anemia during pregnancy is regarded as a substantial risk factor for maternal death, premature birth, and inadequate newborn and child development (Allen, 2005). Therefore, women, particularly during their pregnancies, are provided with iron supplementation tablets.

Dietary calcium intake among the women in the LMICs included in this study could not meet their EAR. This finding is consistent with the previous studies in both developing countries (Harika et al., 2017; Lee et al., 2013; Torheim et al., 2010) and developed countries (Bellows et al., 2020), which indicated that dietary intake of calcium was inadequate among both NPNL and PW. The deficiencies of calcium might cause pre‐eclampsia, which, together with eclampsia, contributes to maternal and perinatal morbidity and mortality. This risk could be substantially prevented through adequate dietary calcium intake before and in early pregnancy (Hofmeyr et al., 2007). Hence, for both women who are pregnant and those who are childbearing age, the consumption of calcium‐rich foods like milk and dairy products might be encouraged.

Concurrent inadequate intake of dietary iron, zinc, and calcium and dependence on plant‐based diets might lead to alarming the bioavailability of these minerals due to the inhibitory effect of phytate (Al Hasan et al., 2016; Schlemmer et al., 2009). Because of the substantial amount of cereals and legumes in traditional meals in developing nations, phytate intake is quite high (Gibson et al., 2018; Schlemmer et al., 2009). Moreover, the molar ratios of dietary phytate‐to‐iron, phytate‐to‐calcium, and phytate‐to‐zinc are used to estimate iron and zinc bioavailability and to define dietary iron, calcium, and zinc requirements based on diet type (Gibson et al., 2018).

In addition, the current review also shows inadequate intake of vitamin A and folate. These findings are synchronized with a recent review among reproductive‐aged and pregnant women in four LMICs, Ethiopia, Kenya, Nigeria, and South Africa. They found that the prevalence of vitamin A, zinc, and folate deficiencies ranged 4%–22%, 34%, and 46% in WRA, while it ranged 21%–48%, 46%–76%, and 3%–12% in PW, respectively (Harika et al., 2017). Insufficient dietary intake of folate was also found among pregnant women of other developing countries (Lee et al., 2013; Torheim et al., 2010). Evidence suggests that deficiency of folate before conception and inadequate intake during pregnancy cause neural tube defects leading to stillbirths (Imdad et al., 2011). Although women are provided with iron and folic acid (IFA) supplementation during pregnancy, to get the optimal benefit, it should be underscored to provide adequate IFA to women before conception as well through dietary modification and supplementation. Community‐based counseling of women of reproductive age on the importance of dietary diversity and micronutrient intake should be addressed in order to promote greater consumption of micronutrient‐rich green leafy vegetables, whole grain breads/cereals, oily fish, eggs, and fortified food products. Supplementation of IFA and multiple micronutrients to the women during preconception and conception could be another approach to combat the situation where requires. Biofortification of plan‐based foods with desirable micronutrients could be an effective approach in combating inadequacy of dietary micronutrients and subsequent micronutrient deficiency‐related malnutrition in LMICs (Ofori et al., 2022).

The study has some strengths as well as few unavoidable limitations. The use of valid approaches to assess dietary diversity and micronutrient intake in all the studies gives this review more strength. Most studies assessed dietary diversity according to the FAO recommended MDD‐W guidelines and micronutrient intake based on 24‐h dietary recall data. However, this review should be considered with some inevitable limitations. Single 24‐h recall method might not represent the usual dietary intake of various nutrients. In summarizing micronutrient intake, only the mean/median intake of various nutrients was compared with ICMR recommended EAR cut‐point without giving much attention to the individual regional variations in the requirements, which might contribute to misconstruing the results. Moreover, there might be a regional effect in the findings since more studies were from Asian regions compared to Africa and Latin America. The study's findings could not be generalized to LMICs since several studies included in this review were not nationally representative. Moreover, this review included 21 eligible studies on dietary diversity from 15 different LMICs and 14 studies on dietary micronutrient intake from 10 different LMICs. Thus, the coverage of LMICs might not be comprehensive. In addition, the study did not consider the season (e.g., dry/rainy or harvest/preharvest seasons), which might influence dietary patterns and intake of vitamins and minerals. Since dietary pattern varies with factors such as age, life stage, season, ethnicity, socio‐economic status, and region, further research is warranted to report the intake of micronutrients at the subpopulation level to find the determinants of micronutrients inadequacy among the women of LMICs. Additionally, the study did not include conference papers or theses, which may have offered more context for the relevant findings. Only two electronic databases (PubMed and Google Scholar) were used in the review to find relevant articles. However, only Google Scholar can offer relevant original articles found by searching various databases for systematic reviews and meta‐analyses (Jean‐François et al., 2013). Thus, it is anticipated that using PubMed and Google Scholar to search for literature can provide adequate coverage of relevant articles for this study.

## 5 CONCLUSION

The available studies in this review indicate a deplorable situation in dietary diversity and micronutrient intake among WRA from most of the LMICs, notably South Africa, Zambia, and Lebanon. Multiple micronutrient intakes, especially vitamin A, thiamin, riboflavin, folate, vitamin B_12_, iron, zinc, and calcium, did not meet dietary intake recommendations. Nationally representative data of LMICs on dietary diversity and micronutrient intake are needed for exploring the conspicuous insight into these two public health issues. Effective public health measures, including dietary diversification, food fortification with micronutrients, making these foods affordable, and micronutrient supplementation could help improve the dietary quality and intake of optimal micronutrients by women in LMICs.

## AUTHOR CONTRIBUTIONS

**Md. Hafizul Islam:** Conceptualization (equal); data curation (equal); formal analysis (equal); methodology (equal); writing – original draft (equal); writing – review and editing (equal). **Md. Moniruzzaman Nayan:** Writing – original draft (supporting); writing – review and editing (supporting). **Ahmed Jubayer:** Formal analysis (supporting); writing – original draft (supporting); writing – review and editing (supporting). **Md. Ruhul Amin:** Conceptualization (equal); methodology (supporting); supervision (lead); writing – review and editing (lead).

## FUNDING INFORMATION

This study was not funded.

## CONFLICT OF INTEREST STATEMENT

The authors declared no conflicts of interest for any part of this article.

## Data Availability

Data sharing is not applicable to this article as no datasets were generated or analyzed during the current study.
